# Acute hyperglycaemia enhances oxidative stress and aggravates myocardial ischaemia/reperfusion injury: role of thioredoxin-interacting protein

**DOI:** 10.1111/j.1582-4934.2012.01661.x

**Published:** 2013-01-11

**Authors:** Hui Su, Lele Ji, Wenjuan Xing, Wei Zhang, Heping Zhou, Xinhong Qian, Xiaoming Wang, Feng Gao, Xin Sun, Haifeng Zhang

**Affiliations:** a Department of Geriatrics, Xijing Hospital, Fourth Military Medical UniversityXi'an, China; b Department of Physiology, Fourth Military Medical UniversityXi'an, China; c Department of Cardiology, Tangdu Hospital, Fourth Military Medical UniversityXi'an, China; d Department of Cardiac Surgery, Xijing Hospital, Fourth Military Medical UniversityXi'an, China; e Department of Pediatrics, Xijing Hospital, Fourth Military Medical UniversityXi'an, China; f Center of Teaching Experiment, Fourth Military Medical UniversityXi'an, China

**Keywords:** Hyperglycaemia, Txnip, myocardial ischaemia/reperfusion, oxidative stress, p38 mitogen-activated protein kinase, Akt

## Abstract

Hyperglycaemia during acute myocardial infarction is common and associated with increased mortality. Thioredoxin-interacting protein (Txnip) is a modulator of cellular redox state and contributes to cell apoptosis. This study aimed to investigate whether or not hyperglycaemia enhances Txnip expression in myocardial ischaemia/reperfusion (MI/R) and consequently exacerbates MI/R injury. Rats were subjected to 30 min. of left coronary artery ligation followed by 4 hrs of reperfusion and treated with saline or high glucose (HG, 500 g/l, 4 ml/kg/h intravenously). *In vitro* study was performed on cultured rat cardiomyocytes subjected to simulated ischaemia/reperfusion (SI/R) and incubated with HG (25 mM) or normal glucose (5.6 mM) medium. *In vivo* HG infusion during MI/R significantly impaired cardiac function, aggravated myocardial injury and increased cardiac oxidative stress. Meanwhile, Txnip expression was enhanced whereas thioredoxin activity was inhibited following HG treatment in ischaemia/reperfusion (I/R) hearts. In addition, HG activated p38 MAPK and inhibited Akt in I/R hearts. In cultured cardiomyocytes subjected to SI/R, HG incubation stimulated Txnip expression and reduced thioredoxin activity. Overexpression of Txnip enhanced HG-induced superoxide generation and aggravated cardiomyocyte apoptosis, whereas Txnip RNAi significantly blunted the deleterious effects of HG. Moreover, inhibition of p38 MAPK or activation of Akt markedly blocked HG-induced Txnip expression in I/R cardiomyocytes. Most importantly, intramyocardial injection of Txnip siRNA markedly decreased Txnip expression and alleviated MI/R injury in HG-treated rats. Hyperglycaemia enhances myocardial Txnip expression, possibly through reciprocally modulating p38 MAPK and Akt activation, leading to aggravated oxidative stress and subsequently, amplification of cardiac injury following MI/R.

## Introduction

Hyperglycaemia commonly occurs in patients with acute myocardial infarction and is associated with increased risk of mortality and poor outcome [1, 2]. Although evidence from our laboratory and others has demonstrated that hyperglycaemia exacerbates myocardial vulnerability to ischaemia/reperfusion (I/R) [3, 4], the underlying mechanisms remain to be identified.

Strong evidence exists that increased oxidative stress, which oxidizes biological macromolecules and impairs cell functions, is a major pathogenic factor in I/R injury and diabetes [5, 6]. Growing evidence has also shown that hyperglycaemia promotes excess generation of highly reactive oxygen species (ROS) and causes oxidative stress which further exacerbates the development and progression of diabetes and its complications [7]. Thus, oxidative stress is a reasonable link between hyperglycaemia and myocardial vulnerability to I/R. However, the ultimate culprit factor(s) which mediated hyperglycaemia-induced oxidative stress and aggravated myocardial ischaemia/reperfusion (MI/R) injury remain unknown.

As an intracellular ROS-scavenging system, thioredoxin (Trx) system (Trx, Trx reductase, and NADPH) is a major antioxidative system which contributes to cellular redox balance and controls ROS formation. Trx reduces ROS through an interaction with the redox-active centre of Trx to form a disulphide bond, which in turn can be eliminated by Trx reductase and NAPDH [8]. Thioredoxin-interacting protein (Txnip), formerly known as vitamin D_3_-up-regulated protein-1 or thioredoxin binding protein-2, inhibits Trx anti-oxidative function by binding to its redox-active cysteine residues. Overexpression of Txnip inhibits the reducing activity of Trx and thereby can modulate the cellular redox state and promote oxidative stress [9, 10], and this Txnip/Trx axis has an important role in the preservation of cellular viability [11]. In addition, experimental evidence has indicated that hyperglycaemia and diabetes could induce Txnip expression and decrease Trx activity [12]. However, direct evidence to support a causative role of Txnip in hyperglycaemia-aggravated MI/R injury is not currently available.

Hyperglycaemia can increase the generation of free radicals and proinflammatory cytokines, further impair activation of Akt and increase apoptosis in cultured cardiomyocytes [13]. Our previous study has also indicated that acute hyperglycaemia during ischaemia impairs Akt activation in I/R myocardium [3]. In addition, previous studies have indicated that lack of Txnip induces Akt signalling and improves insulin resistance [14, 15]. All these suggest a close association between Akt and Txnip in hyperglycaemia. On the other hand, p38 mitogen-activated protein kinase (p38 MAPK) is an important stress signalling molecule and is involved in the regulation of many cellular functions. Patients with insulin resistance and/or type 2 diabetes have high levels of plasma free fatty acids, inflammatory cytokines, and/or glucose which can activate p38 MAPK [16]. Once p38 MAPK pathway is activated, it is capable of damaging Trx system that further increases intracellular ROS level [17]. Thus, we specially focus on the roles of Akt and p38 MAPK in hyperglycaemia-related Txnip expression in I/R hearts.

The aims of this study were to (1) determine the role of Txnip in hyperglycaemia-aggravated MI/R injury; (2) investigate the underlying mechanism with a special focus on Akt and p38 MAPK.

## Materials and methods

The experiments were performed in adherence with the National Institutes of Health Guidelines for the Use of Laboratory Animals and were approved by the Fourth Military Medical University Committee on Animal Care.

### Experimental protocol

Adult male Sprague-Dawley rats were fasted overnight and anesthetized through intraperitoneal administration of 60 mg/kg pentobarbital sodium. Myocardial ischaemia was produced by exteriorizing the heart with a left thoracic incision followed by making a slipknot (6-0 silk) around left anterior descending (LAD) coronary artery, as previously described [18]. A microcatheter was inserted into LV through right carotid artery to measure the LV pressure. The artery pressure was measured by right femoral artery intubation. Intravenous infusion was executed through left external jugular vein. Hemodynamic data were continuously monitored on a polygraph and simultaneously digitized by using a computer interfaced with an analogue-to-digital converter. Blood samples were drawn from caudal vein before ischaemia, 30 min. after ischaemia, 2 and 4 hrs after reperfusion, respectively, to measure blood glucose levels by a glucose meter (Life-Scan, Milpitas, CA, USA).

After 30 min. of ischaemia, the slipknot was released and the myocardium was reperfused for 4 hrs. Rats randomly received one of the following solutions by intravenous infusion at a rate of 4 ml/kg/h (*n* = 8/group): (1) MI/R+V (V, vehicle): saline throughout the whole ischaemia and reperfusion period, beginning 5 min. before ischaemia; (2) MI/R+HG: high glucose (HG) throughout the whole ischaemia and reperfusion period (glucose 500 g/l). Sham-operated control rats (Sham MI/R) underwent the same surgical procedures with the exception of left anterior descending coronary artery occlusion. Hearts were excised at the end of reperfusion and the tissue from the area-at-risk was harvested. The area-at-risk was delineated from the area-not-at-risk by visualization of pallor upon transient LAD occlusion, immediately prior to tissue dissection. In separate rats, hearts were excised to determine myocardial infarct size.

### Determination of myocardial infarction and apoptosis

At the end of 4-hr reperfusion, myocardial infarction was determined by means of a double-staining technique and a digital imaging system (infarct area/area-at-risk ×100%) [3]. Myocardial apoptosis was analysed by TUNEL (terminal deoxynucleoti-dyl transferase dUTP nick end labelling) assay using an *in situ* cell death detection kit (Roche Molecular Biochemicals, Mannheim, Germany). TUNEL staining for apoptotic cell nuclei and 4′,6-diamino-2-phenylindole staining for all myocardial cell nuclei and α-sarcomeric actin staining for cardiomyocytes as described previously [3]. The index of apoptosis was expressed by number of apoptotic myocytes/the total number of myocytes counted ×100%. The caspase-3 activity of cardiomyocytes was measured by using caspase colorimetric assay kits (Chemicon International, Temecula, CA, USA), as described in our previous study [5].

### Determination of plasma creatine kinase and lactate dehydrogenase

Blood samples (1 ml) were drawn at 4 hrs after reperfusion. Plasma creatine kinase (CK) and lactate dehydrogenase (LDH) activities were measured spectrophotometrically (Beckman DU 640) in a blinded manner. All measurements were assayed in duplicate.

### Quantification of superoxide production

Superoxide production in tissue or cells was measured by lucigenin-enhanced chemiluminescence as described previously [19, 20]. Superoxide production was expressed as relative light units (RLU) per second per milligram heart weight (RLU/mg/s).

### Determination of tissue malondialdehyde and superoxide dismutase

The malondialdehyde (MDA) level and activities of antioxidant enzyme superoxide dismutase (SOD) in heart homogenates were determined spectrophotometrically as previously described [21].

### Cell preparation and *in vitro* simulated ischaemia/reperfusion model

Primary cultured neonatal rat cardiomyocytes from 1-day-old Sprague-Dawley rats were employed. The hearts were rapidly excised, minced and dissociated with 0.1% trypsin and 0.03% collagenase. The dispersed cells were then plated at a field density of 2 × 10^5^ cells/cm^2^ on 60-mm culture dishes with DMEM supplemented with 10% foetal bovine serum (FBS), 100 units/ml penicillin/streptomycin, and 0.1 mM 5-bromo-2-deoxyuridine to inhibit fibroblast proliferation. Simulated ischaemia/reperfusion (SI/R) was performed as previously described [22]. In brief, cardiomyocytes were exposed to glucose-free serum-free culture medium and transferred into a Modular Incubator Chamber (Billumps-Rothenberg) flushed with 5% CO_2_ and 95% N_2_ for 2 hrs of hypoxia at 37°C. After hypoxia, the culture medium was replaced with fresh oxygenated normal or high-glucose cultured medium, and the dishes were transferred to a normoxic incubator (95% air-5% CO_2_) for 4 hrs of reoxygenation. All cells were starved with serum-free medium for 12 hrs before normoxia or hypoxia treatment. Glucose concentrations in HG and control medium were 25 mM and 5.6 mM respectively.

### Trx activity assay

Trx activity was measured using the insulin disulphide reduction assay as previously described [12], and expressed as nicotinamide adenine dinucleotide phosphate oxidized (μ mol) per minute per milligram of protein.

### Real time PCR

Txnip gene expression was analysed by real time PCR (FTC-3000 qPCR System, Funglyn Biotech Inc, Shanghai, China) using specific oligonucleotides [12]: Txnip, 5′-CAAGTTCGGCTTTGAGCTTC-3′ (sense) and 5′-GCCATTGGCAAGGTAAGTGT-3′ (antisense); Trx, 5′-GCTGATCGAGAGCAAGGAAG-3′ (sense) and 5′-TCAAGGAACACCACATTGGA-3′ (antisense). GAPDH was used as the internal control, 5′-GGACCTGACCTGCCGTCTAG-3′ (sense) and 5′-TAGCCCAGGATGCCCTTGAG-3′ (antisense).

### Western blot analysis

The expressions of Txnip, Trx, Akt, p38 MAPK and the phosphorylation of Akt and p38 MAPK were measured using Western blot as described previously [23]. Protein content was determined with BCA protein assay and protein samples were separated by electrophoresis on SDS-PAGE and transferred to a polyvinylidene difluoride membrane. The membranes were blocked with 5% milk and incubated overnight with the appropriate primary antibodies respectively [anti-Txnip, anti-Trx (Santa Cruz Biotechnology, Santa Cruz, CA, USA), anti-gp91^phox^, anti-phospho-(p)-Akt, anti-Akt (Cell Signaling Technology, Beverly, MA, USA), anti-p-p38 MAPK, anti-p38 MAPK (Abcam, Cambridge, UK)], followed by incubation with the corresponding secondary antibodies. The blots were visualized with ECL-plus reagent. Beta-actin was used as the internal loading control.

### Adenovirus infection

Adenoviral vectors for overexpression of Trx (AdTrx) and Txnip (AdTxnip) were used as previously described [11]. Viruses were propagated in 293 cells and kept at −80°C with a titre of 10^11–12^ pfu/ml before use. Gene transfer with adenovirus encoding GFP was used as an internal control. Cardiomyocytes plated at a density of 0.5 to 1 × 10^5^/cm^2^ were infected by adenovirus at 50 multiplicity of infection (moi) for 2 hrs at 37°C in a humidified, 5% CO_2_ incubator. Subsequently, the cells were cultured in serum-free DMEM media for an additional 24 hrs before processing.

### Transfection of siRNAs

Cardiomyocytes were allowed to attach for 16–24 hrs prior to transfection. Double-stranded RNAi for selective silencing of Txnip (aaacagaccttggactacttt) with a final concentration of 100 nM was transfected into cells (FuGENE Reagent, Roche Applied Science, Mannheim, Germany) according to the manufacturer's instructions. After transfection, cells were incubated with HG for Txnip expression. Scrambled RNAi was used as control. Knockdown of gene expression was identified by Western blot analysis 48 hrs after transfection.

### Intramyocardial injection of Txnip siRNA *in vivo*

Txnip specific small interfering RNA or scrambled siRNA (20 μg diluted in 40 μl vivo-jetPEI™ and 10% glucose mixture) were injected into the apex and anterolateral wall of the heart with a 30-gauge needle in rats. After 48 hrs of siRNA injection, the rats were subjected to 30 min. of ischaemia and 4 hrs of reperfusion, receiving HG throughout the whole ischaemia and reperfusion period (glucose 500 g/l, 4 ml/kg/h intravenously). At the end of reperfusion, hearts were excised for the determinations.

### Statistical analysis

All values are presented as means ± SEM. Differences were compared by anova followed by Bonferroni correction for post hoc *t* test, where appropriate. Probabilities of <0.05 were considered to be statistically significant. All the statistical tests were performed with the GraphPad Prism software version 5.0 (GraphPad Software, San Diego, CA, USA).

## Results

### Hyperglycaemia aggravated I/R-induced cardiac dysfunction

**Fig. 1:**
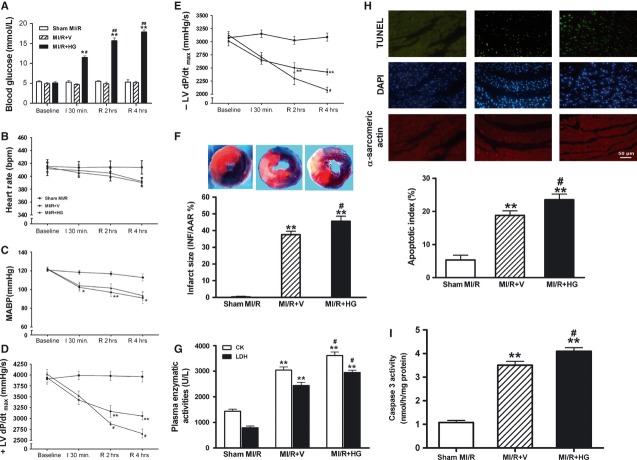
Hyperglycaemia decreased cardiac functions and increased myocardial injury in rats subjected to myocardial ischaemia/reperfusion. (**A**) Blood glucose levels; (**B**) Heart rate; (**C**) MABP, mean arterial blood pressure; (**D**) +LVdP/dt_max_, the instantaneous first derivation of left ventricle pressure; (**E**) -LVdP/dt_max_. MI/R, myocardial ischaemia/reperfusion (30 min./4 hrs); (**F**) Top: representative photographs of heart sections. Blue-stained portion indicates non-ischaemic, normal region; red-stained portion, ischaemic/reperfused but not infarcted region; and negative-stained portion, ischaemic/reperfused infarcted region. Bottom: myocardial infarct size expressed as percentage of area-at-risk (AAR); (**G**) Plasma creatine kinase (CK) and lactate dehydrogenase (LDH) levels; (**H**) Top: representative photomicrographs of *in situ* detection of apoptotic myocytes by TUNEL staining. Green fluorescence shows TUNEL-positive nuclei; blue fluorescence shows nuclei of total cardiomyocytes; Red fluorescence shows cardiomyocytes; Original magnification×400; Bottom: percentage of TUNEL-positive nuclei in heart tissue sections; (**I**) Myocardial caspase-3 activity. MI/R, myocardial ischaemia/reperfusion (30 min./4 hrs); Sham MI/R, sham-operated; V, vehicle; HG, high glucose; I, ischaemia; R, reperfusion. Values presented are means ± SEM. *n* = 8/group. *P < 0.05, **P < 0.01 *versus* Sham MI/R, ^*#*^*P* < 0.05, ^*##*^*P* < 0.01 *versus* MI/R+V.

As shown in Fig. 1A, no significant differences in blood glucose levels were observed among all groups under baseline conditions. MI/R has no effect on blood glucose while high glucose infusion during MI/R significantly increased blood glucose compared with those in vehicle.

No significant differences were observed in systemic hemodynamics among all groups under baseline conditions. There were no significant differences in heart rate among all groups during ischaemia or reperfusion period although blood pressure was significantly decreased in both groups following ischaemia. MI/R significantly impaired cardiac functions as evidenced by decreased + LVdP/dt_max_ and −LVdP/dt_max_ after 4 hrs of reperfusion (*P* < 0.01). Compared with vehicle-treated group, HG further reduced ±LVdP/dt_max_ by 13.2 and 14.1% respectively (*P* < 0.05, Fig. 1D and E). These data demonstrated that hyperglycaemia during MI/R markedly decreased cardiac function.

### Hyperglycaemia exacerbated I/R-induced myocardial injury

Thirty minutes of ischaemia and 4 hrs of reperfusion resulted in myocardial injury, as evidenced by increased infarct size (Fig. 1F), plasma CK/LDH activities (Fig. 1G) and myocardial apoptosis (Fig. 1H and I). HG treatment further elevated MI/R-induced deleterious effects (all *P* < 0.05). These results provided direct evidence that hyperglycaemia during MI/R exacerbated myocardial injury.

### Hyperglycaemia increased oxidative stress in I/R hearts

**Fig. 2:**
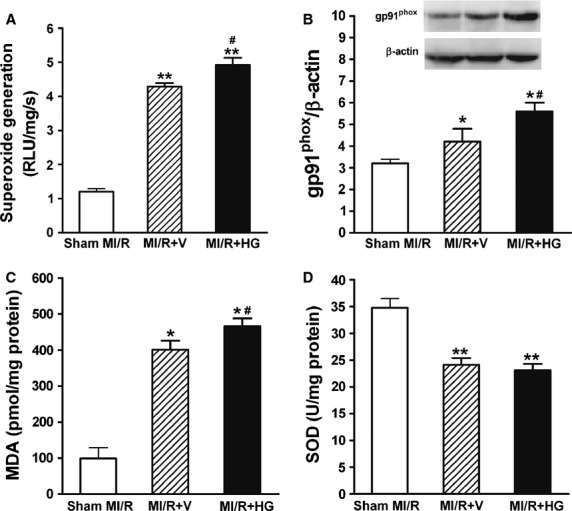
Hyperglycaemia enhanced oxidative stress in rats subjected to myocardial ischaemia/reperfusion. (**A**) Cardiac superoxide generation; (**B**) gp91^phox^ expression. Top images: representative blots; (**C**) Myocardial malondialdehyde (MDA) contents; (**D**) Myocardial superoxide dismutase (SOD) contents. MI/R, myocardial ischaemia/reperfusion (30 min./4 hrs); Sham MI/R, sham-operated; V, vehicle; HG, high glucose. Values presented are means ± SEM. *n* = 8/group. *P < 0.05, **P < 0.01 *versus* Sham MI/R, ^*#*^*P* < 0.05 *versus* MI/R+V.

As seen in Fig. 2A, compared with sham-operated hearts, I/R myocardium showed a significant increase in superoxide content (*P* < 0.01), and HG treatment further enhanced superoxide accumulation (*P* < 0.05 *versus* MI/R+V). Afterwards, we determined gp91^phox^ expression, a major component of NADPH oxidase that is the most important superoxide-producing enzyme in the ischaemic reperfused heart. As expected, HG infusion markedly increased MI/R-stimulated gp91^phox^ expression (*P* < 0.05, Fig. 2B). In addition, MDA was observed as a biomarker to measure the level of oxidative stress. There was a robust increase in MDA production in I/R hearts compared with the sham objects, and treatment with HG amplified this harmful effect (Fig. 2C). In contrast, antioxidant enzymes SOD content in cardiac tissue was also reduced in MI/R rats with or without HG infusion (Fig. 2D). These results demonstrated that hyperglycaemia stimulated superoxide overproduction and increased oxidative stress in I/R hearts.

### Hyperglycaemia stimulated Txnip expression and inhibited Trx activity in I/R hearts

**Fig. 3:**
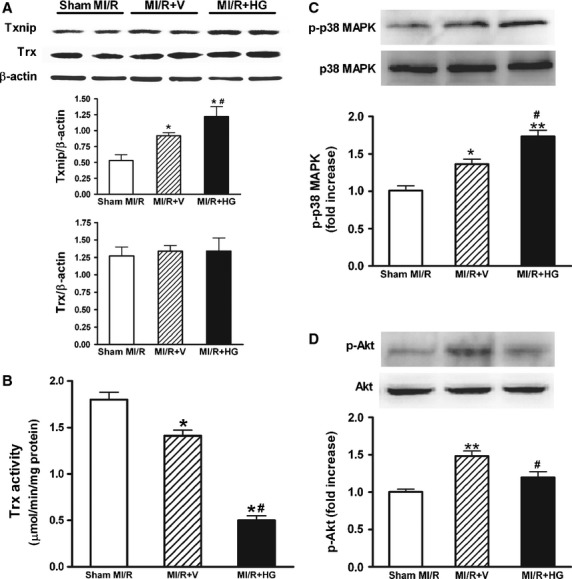
Hyperglycaemia stimulated Txnip expression and inhibited Trx activity in rats subjected to myocardial ischaemia/reperfusion. (**A**) Txnip and Trx expression; (**B**) Myocardial Trx activity; (**C**) Phosphorylation of p38 MAPK; (**D**) Phosphorylation of Akt. Top images of A, C and D: representative blots; MI/R, myocardial ischaemia/reperfusion (30 min./4 hrs); Sham MI/R, sham-operated; V, vehicle; HG, high glucose. Values presented are means ± SEM. *n* = 6–8/group. *P < 0.05 *versus* Sham MI/R, ^*#*^*P* < 0.05 *versus* MI/R+V.

As Txnip accumulation is known to promote oxidative stress, we next determined myocardial Txnip expression in hyperglycaemia-treated I/R hearts. As summarized in Fig. 3A, both MI/R with vehicle and HG-stimulated myocardial Txnip expression. Furthermore, HG-treated hearts showed a significant increase in Txnip content compared with those in vehicle. These data suggested that MI/R-induced Txnip was enhanced after HG treatment. As Txnip is a physiological inhibitor of Trx, the consequences of Txnip overproduction by I/R and HG have been studied by assessing Trx activity. Although no significant difference in Trx expression was observed among all groups, Trx activity was significantly inhibited following MI/R (*P* < 0.05, Fig. 3B), and HG infusion further down-regulated I/R-reduced Trx activity (*P* < 0.05). These results indicated that hyperglycaemia could further stimulate MI/R-induced Txnip expression and subsequently inhibit Trx activity.

### Hyperglycaemia activated p38 MAPK and inhibited Akt in I/R hearts

To examine the potential signalling pathways involved in HG and/or I/R-elicited cardiac injury, we examined phosphorylation of cellular stress-sensitive kinase p38 MAPK and the anti-apoptotic factor Akt. As shown in Fig. 3C and D, there was no significant difference in p38 MAPK or Akt expression among all groups. However, phosphorylation of p38 MAPK and Akt was increased following MI/R. Compared with MI/R, HG infusion further stimulated p38 MAPK activation. In contrary, MI/R-induced Akt activation was markedly inhibited after HG treatment. These results indicated that HG treatment activated the stress kinase p38 MAPK whereas inhibited survival Akt signalling in I/R hearts.

### Txnip mediated hyperglycaemia-aggravated oxidative stress and SI/R injury *in vitro*

**Fig. 4:**
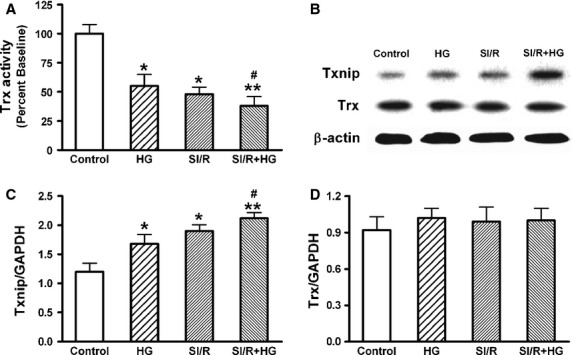
Hyperglycaemia stimulated Txnip expression and inhibited Trx activity in stimulated ischaemia/reperfusion cardiomyocytes. (**A**) Myocardial Trx activity expressed as percentage of baseline; (**B**) Western blots showing Txnip and Trx protein expression; (**C**) Txnip mRNA expression; (**D**) Trx mRNA expression. Control, normal culture condition; SI/R, simulated ischaemia/reperfusion; HG, high glucose. Values presented are means ± SEM. *n* = 6/group. *P < 0.05, **P < 0.01 *versus* Control, ^*#*^*P* < 0.05 *versus* SI/R.

Our *in vivo* experimental results demonstrated that treatment with HG significantly increased Txnip expression, inhibited Trx activity, stimulated superoxide production and thus aggravated myocardial injury following MI/R. To obtain more evidence to support a causative link between Txnip and HG-aggravated oxidative stress and myocardial injury, an additional study was performed using cultured neonatal rat cardiomyocytes. As illustrated in Fig. 4A, exposing cardiomyocytes to HG or SI/R significantly decreased Trx activity (*P* < 0.05). Moreover, HG plus SI/R showed a further reduction in Trx activity compared with SI/R group (*P* < 0.05). No significant difference was observed in the expression of Trx mRNA or protein levels among all groups (Fig. 4B and D). Importantly, increased expression of Txnip was found in HG or SI/R groups, as evidenced by elevated protein and mRNA levels. Cardiomyocytes incubated with HG following SI/R showed a higher Txnip expression (Fig. 4B and C). These results demonstrated that I/R-induced Txnip expression was up-regulated after incubation with HG *in vitro*.

**Fig. 5:**
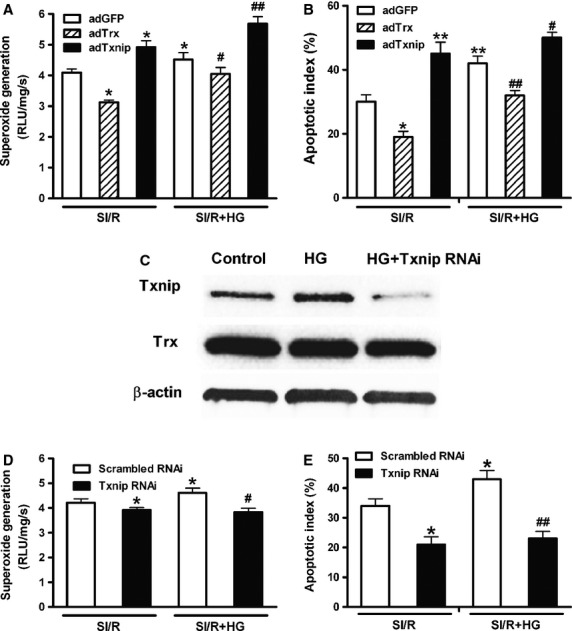
Hyperglycaemia promoted oxidative stress and aggravated ischaemia/reperfusion cardiomyocyte injury through regulation of Txnip expression. (**A**) Cardiac superoxide generations after adenoviral gene transfer of Txnip or Trx in SI/R with or without hyperglycaemia; Cells were infected with adenoviral vectors for overexpression of Txnip, Trx, or GFP alone (controls) for 48 hrs before SI/R. (**B**) Percentage of apoptotic cells as assessed by TUNEL staining. (**C**) Western blots showing Txnip and Trx protein expression after Txnip RNAi. (**D**) Cardiac superoxide generations after gene silencing of Txnip in SI/R with or without hyperglycaemia. Cells were transfected with Txnip silencing or scrambled RNAi for 48 hrs before SI/R. (**E**) Percentage of apoptotic cells as assessed by TUNEL staining. Control, normal culture condition; SI/R, simulated ischaemia/reperfusion; HG, high glucose. Values presented are means ± SEM. *n* = 6/group. *P < 0.05, **P < 0.01 *versus* SI/R+adGFP (or Scrambled RNAi); ^#^*P* < 0.05, ^##^*P* < 0.01 *versus* SI/R+HG+adGFP (or Scrambled RNAi).

To directly clarify the critical role of Txnip in HG-exacerbated SI/R injury, we investigated the effects of up-regulating or down-regulating Txnip expression on superoxide generation and cell apoptosis. Overexpression of Txnip resulted in significant increased cellular levels of superoxide and cardiomyocyte apoptosis following SI/R (both *P* < 0.05). Compared with cardiomyocytes treated with normal glucose, HG incubation during I/R markedly stimulated ROS production and cell apoptosis, and Txnip overexpression exhibited higher ROS production and cardiomyocyte injury. In contrast, adenoviral gene transfer of Trx strongly inhibited the increase in superoxide and apoptosis during SI/R, and blocked the additional deleterious effects of HG incubation (Fig. 5A and B). Moreover, Txnip RNAi significantly inhibited the hyperglycaemia-increased Txnip protein level although there were no changes in Trx expression (Fig. 5C). Consistently, gene knockdown of Txnip significantly reduced SI/R-induced oxidative stress and apoptosis in cardiomyocytes treated with normal or high glucose (Fig. 5D and E). All these data demonstrated that Txnip contributes to hyperglycaemia-induced oxidative stress and cell injury in I/R cardiomyocytes.

### Hyperglycaemia regulated Txnip through p38 MAPK and Akt

**Fig. 6:**
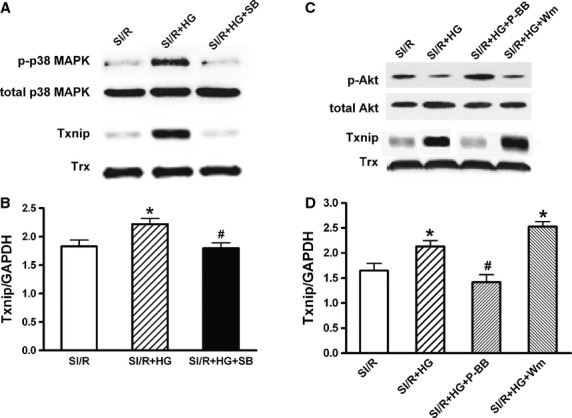
Hyperglycaemia increased Txnip through reciprocally regulating p38 MAPK and Akt. (**A**) Western blots showing p38 MAPK phosphorylation and expressions of total p38 MAPK, Txnip and Trx in I/R cardiomyocytes receiving different treatments. (**B** and **D**) Txnip mRNA expression in cardiomyocytes following different treatments. (**C**) Western blots showing Akt phosphorylation and expressions of total Akt, Txnip and Trx in I/R cardiomyocytes receiving different treatments. SI/R, simulated ischaemia/reperfusion; HG, high glucose; SB, p38 MAPK inhibitor SB239063; P-BB, platelet-derived growth factor PDGF-BB, Akt agonist; Wm, wortmannin, PI3-kinase inhibitor. Values presented are means ± SEM. *n* = 6/group. *P < 0.05 *versus* SI/R, ^*#*^*P* < 0.05 *versus* SI/R+HG.

Our *in vivo* study demonstrated that HG treatment activated p38 MAPK and inhibited Akt. To determining whether HG-stimulated Txnip expression through altering the two signalling pathways, pharmacological inhibitors or agonists were used. As seen in Fig. 6A, there was no significant difference in p38 MAPK expression among all groups. HG incubation significantly stimulated p38 MAPK activation, which was markedly inhibited by p38 inhibitor. Similarly, there was a significant increase in the expression of Txnip protein (Fig. 6A) and mRNA (Fig. 6B) after HG treatment, whereas p38 inhibitor SB239063 abolished these effects. These data suggested that HG-induced p38 MAPK activation was essential for Txnip expression. Our further results revealed that although Akt expression had no changes, HG treatment effectively reduced Akt phosphorylation (Fig. 6C). Co-incubation with platelet-derived growth factor (PDGF-BB, 4 ng/ml) was aimed to increase Akt phosphorylation whereas wortmannin (100 nM, PI3-kinase inhibitor) was treated to inhibit Akt activation. Interestingly, co-incubation with PDGF-BB markedly reduced HG-induced Txnip protein (Fig. 6C) and mRNA (Fig. 6D) expressions following SI/R whereas pre-treatment with wortmannin significantly increased Txnip expression. The data revealed that Akt activation inhibited Txnip expression, and in turn, its inactivation in hyperglycaemia promoted the expression of Txnip. Taken together, these findings suggested involvement of p38 MAPK and Akt in hyperglycaemia-regulated Txnip expression.

### Suppressing Txnip with Txnip siRNA markedly alleviated MI/R injury in HG-treated rats

**Fig. 7:**
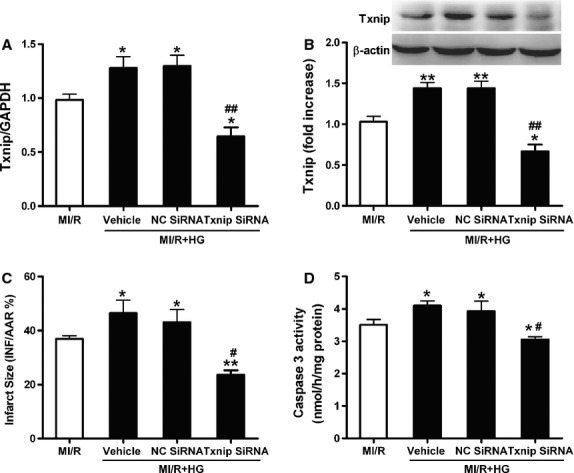
Suppressing Txnip with Txnip siRNA alleviated myocardial injury in high glucose-treated rats following 30 min. of ischaemia and 4 hrs of reperfusion. (**A**) Txnip mRNA expression; (**B**) Txnip protein expression, Top images: representative blots; (**C**) myocardial infarct size expressed as percentage of area-at-risk (AAR); (**D**) Myocardial caspase-3 activity. MI/R, myocardial ischaemia/reperfusion (30 min./4 hrs); HG, high glucose; NC, negative control. Values presented are means ± SEM. *n* = 4–5/group. *P < 0.05, **P < 0.01 *versus* MI/R, ^#^*P* < 0.05, ^##^*P* < 0.01 *versus* NC SiRNA.

Having demonstrated that hyperglycaemia reciprocally modulated p38 MAPK and Akt activation and thus enhanced myocardial Txnip expression, ultimately aggravating SI/R injury *in vitro*, we next used a genetic approach to reduce Txnip *in vivo* to obtain more solid evidence to support a causative role of Txnip and increased myocardial injury. After 48 hrs of intramyocardial siRNA (Txnip siRNA or scrambled siRNA) injection, the rats were subjected to MI/R as described above and treated with HG (500 g/l, 4 ml/kg/h intravenously). As summarized in Fig. 7A and B, cardiac expression of Txnip mRNA and protein level were both decreased after Txnip siRNA injection in HG-treated I/R myocardium (*P* < 0.01). We next determined whether or not the reduction in Txnip could reverse MI/R injury following HG infusion. As shown in Fig. 7C and D, myocardial injury in Txnip siRNA-treated hearts was markedly alleviated compared with vehicle or scrambled siRNA-injected hearts, as evidenced by decreased myocardial infarct size and caspase-3 activity (*P* < 0.05). Therefore, reduction in Txnip in HG-treated hearts increased the tolerance of these hearts to ischaemic injury.

## Discussion

The major findings from this study are as follows. First, we have demonstrated that hyperglycaemia increases I/R-induced myocardial Txnip expression *in vivo*, accompanying with increased oxidative stress and myocardial injury. Second, our *in vitro* study has provided the first evidence that Txnip is one of the ultimate culprit factors which mediate hyperglycaemia-aggravated oxidative stress and ischaemic injury, and the hyperglycaemia-increased Txnip expression is, at least in part, due to activation of p38 MAPK and inhibition of Akt. Third and most important, intramyocardial injection of Txnip siRNA markedly decreased Txnip expression and alleviated MI/R injury in HG-treated rats. To the best of our knowledge, this is the first report demonstrating the critical role of Txnip in hyperglycaemia-exacerbated MI/R injury.

Hyperglycaemia with myocardial infarction is associated with an increased risk of in-hospital mortality in patients [2]. Experimental evidence has suggested that diabetic hearts reveal an increased susceptibility to cardiac ischaemia [24, 25]. As stated by previous studies, stress hyperglycaemia was defined as blood glucose ≥10 mmol/l [26, 27], which was associated with higher mortality [28]. In our preliminary study, we found that rats receiving glucose (500 g/l) showed severe hyperglycaemia (≥15 mmol/l), which simulated the stress hyperglycaemia seen in human patients with AMI. In addition, our present study demonstrated that hyperglycaemia aggravated I/R-induced myocardial injury in animals without diabetes [3]. Thus, hyperglycaemia is an established risk factor during myocardial infarction. Of the many theories regarding the development of reperfusion injury, the enhanced generation of highly ROS by the heart during the acute reperfusion phase, including superoxide anion, hydrogen peroxide and hydroxyl radical, is an appealing one that is supported by a large foundation of experimental evidence [6, 29]. In this study, we have demonstrated that hyperglycaemia further promoted MI/R-induced ROS production, MDA content and gp91^phox^ expression (a critical component of NADPH oxidase which is one of the major sources of superoxide anion in the heart). In addition, cardiac SOD content was significantly impaired in I/R hearts with or without HG treatment. The loss of membrane integrity seems to be the major mechanism of free radical-mediated MI/R injury. Also, free radicals may depress Ca^2+^-regulatory mechanism ultimately results in intracellular Ca^2+^ overload and cell death [30]. These data suggest that hyperglycaemia aggravated oxidative stress and reperfusion injury in I/R hearts.

Txnip has a molecular mass of 50 kD and is originally identified in a yeast two-hybrid screen for proteins that bind to Trx. It has been found that Txnip regulates the cellular redox state by binding to and inhibiting Trx in a redox-dependent fashion. In addition, accumulating studies have indicated important roles for Txnip in redox-independent signalling, including metabolic control, regulation of HIF-1 and suppression of proliferation [31]. Moreover, evidence has suggested that Txnip shuttles into the mitochondria in response to oxidative stress, binds to and oxidizes Trx2, thereby reduces Trx2 binding to ASK1 and allowes for ASK1 activation, resulting in induction of the mitochondrial pathway of apoptosis [32]. Hyperglycaemia has been identified as an inducer of Txnip expression in many types of cells including cardiomyocytes [12, 17, 33–35], and Txnip expression is strongly up-regulated in human diabetes [36] and diabetic complications [37], indicating that Txnip is a potential diabetogenic signal. However, few studies have been conducted on hyperglycaemia-regulated Txnip in I/R hearts. Importantly, a very recent study has demonstrated that deletion of Txnip in mice impairs mitochondrial function, but protects the myocardium from ischaemia-reperfusion injury with enhanced anaerobic glycolysis [38], suggesting that Txnip is deleterious to I/R hearts. However, whether Txnip is the ultimate culprit factor mediating hyperglycaemia-increased myocardial vulnerability remains unknown. This study demonstrated for the first time, to our knowledge, that hyperglycaemia promoted I/R-induced myocardial Txnip expression in rat hearts and cardiomyocytes. Moreover, gene silencing of Txnip reduced hyperglycaemia-elevated ROS production and apoptosis in SI/R, whereas Txnip overexpression further enhanced HG-stimulated oxidative stress and aggravated SI/R injury. These data provided the direct evidence that increased Txnip expression plays a key role in excessive ROS production and cell apoptosis induced by hyperglycaemia during I/R. More importantly, our *in vivo* study revealed that intramyocardial injection of Txnip siRNA significantly decreased Txnip expression and thus effectively reduced myocardial infarct size and apoptosis in hyperglycaemia-treated I/R hearts, confirming the critical role of Txnip in hyperglycaemia-aggravated MI/R injury. In contrast, our results indicated that Trx activity was significantly inhibited following MI/R and further decreased in HG group. Notably, adenoviral overexpression of Trx could alleviate these harmful effects of hyperglycaemia during SI/R. Trx is anti-apoptotic and exerts its cardioprotective effects by reducing I/R-induced oxidative/nitrative stress [39]. Recent study has also indicated that Trx overexpression reduces oxidative stress and apoptosis, induces angiogenesis and neovascularization and ultimately prevents the post-ischaemic ventricular remodelling [40]. As Txnip is a well-recognized endogenous inhibitor of Trx, these studies strongly support our conclusion that Txnip overexpression reduced Trx activation, leading to enhanced oxidative stress and aggravating post ischaemic injury.

Different stimuli such as growth factors, inflammatory cytokines or a wide variety of environmental stresses can activate p38 MAPK. Once activated, p38 MAPK targets many important kinases and transcription factors and regulates diverse cellular processes, including gene expression, cell cycle progression, differentiation and apoptosis. Data from this study indicated that HG during I/R significantly increased phosphorylation of p38 MAPK in hearts and cultured cardiomyocytes. Blockage of p38 MAPK pathway by p38 inhibitor in cultured cardiomyocytes not only markedly reduced p38 MAPK activation but also decreased HG-induced Txnip expression. Other studies have also indicated that glucose regulated Txnip at transcription level and p38 MAPK and forkhead box O1 transcriptional factor were involved in the process [12, 17]. These observations favour p38 MAPK as an upstream mechanism for Txnip expression during hyperglycaemia. In addition, Akt is deemed as a key regulator of myocardial function and cell survival. Activation of Akt phosphorylation is reported to stimulate endothelial nitric oxide synthase/nitric oxide pathway and inhibit many pro-apoptotic proteins including BIM, BAX, BAD and p53, ultimately confer protection against myocardium ischaemic injury [41]. Unfortunately, studies from our laboratory and others suggest that hyperglycaemia inhibits Akt signalling and triggers cell apoptosis *via* peroxynitrite-mediated LKB1-dependent PTEN activation [3, 42]. This study also demonstrated that HG significantly reduced Akt phosphorylation in rat hearts and cultured cardiomyocytes. Previous study revealed that nitric oxide, a downstream signalling molecule of Akt, can regulate cellular redox state by suppressing Txnip, and cis-regulatory elements −1127 bp upstream of the start codon mediate this effect [43]. Moreover, experimental evidence exists that Txnip deficiency induces Akt/Bcl-xL signalling and protects against diabetes [14, 44]. These studies suggest that Akt signalling may interplay with Txnip. Data from our *in vitro* study revealed that phosphorylation of Akt by PDGF-BB significantly inhibited HG-induced Txnip expression whereas inactivation of Akt by wortmannin markedly restored Txnip expression, indicating that Akt as a negative regulator of hyperglycaemia-induced Txnip expression. Collectively, in hyperglycaemia state, the detrimental elevated Txnip expression in I/R hearts is, at least in part, due to activation of p38 MAPK and inhibition of Akt. Recent study indicated that activation of mTOR, a highly conserved serine-threonine kinase activated in response to growth factors and nutrients, can be protective against oxidant ischaemic/reperfusion injury in p38- and Akt-dependent manner [45]. Moreover, Txnip is demonstrated as a novel member of the mTOR upstream that acts as a negative regulator in response to stress signals [46]. Thus, it is receivable that in HG condition, p38- and Akt-regulated Txnip modulates signalling from many downstream effector proteins such as Trx, mTOR and eventually aggravates MI/R injury.

Taken together, data from this study have demonstrated that hyperglycaemia enhances myocardial Txnip expression by reciprocally modulating the activation states of p38 MAPK and Akt, contributing to hyperglycaemia-aggravated oxidative stress and ultimately, exacerbating cardiac injury following MI/R. These findings provide some new insights into the underlying mechanisms by which stress hyperglycaemia aggravates myocardial injury in acute myocardial infarction, emphasizing the importance of glycaemic control on cardiovascular disease.
